# C-Cbl negatively regulates TRAF6-mediated NF-*κ*B activation by promoting K48-linked polyubiquitination of TRAF6

**DOI:** 10.1186/s11658-019-0156-y

**Published:** 2019-05-14

**Authors:** Hyun-Duk Jang, Hye Zin Hwang, Hyo-Soo Kim, Soo Young Lee

**Affiliations:** 1 National Leading Laboratory for Stem Cell Research, Seoul National University College of Medicine, Seoul, South Korea; 2 Korea Research-Driven Hospital, Biomedical Research Institute, Seoul National University Hospital, Seoul, South Korea; 3 Strategic Center of Cell & Bio Therapy, Seoul National University Hospital, Seoul, South Korea; 4 Department of Biotechnology, The Catholic University of Korea, Bucheon, South Korea; 5 Cardiovascular Center & Department of Internal Medicine, Seoul National University Hospital, Seoul, South Korea; 6 Department of Life Science and the Research Center for Cellular Homeostasis, Ewha Woman’s University, Seoul, South Korea

**Keywords:** Tumor necrosis factor receptor-associated factor 6, Ubiquitin, E3 ligase, C-Cbl

## Abstract

**Background:**

In its RING domain, tumor necrosis factor receptor-associated factor 6 (TRAF6) has ubiquitin E3 ligase activity that facilitates the formation of lysine 63-linked polyubiquitin chains. This activity is required to activate nuclear factor *κ*-light-chain-enhancer of activated B cells (NF-*κ*B) and plays an important role in the IκB kinase (IKK) complex.

**Methods:**

An in vitro ubiquitination assay was used to establish whether c-Cbl could promote TRAF6 ubiquitination. We assessed direct binding and performed fine mapping between c-Cbl and TRAF6 based on the results of an immunoprecipitation assay with cultured 293 T cells. The luciferase reporter assay was applied to establish if c-Cbl-mediated ubiquitination affected NF-*κ*B activation after stimulus from various TRAF-mediated signals: tumor necrosis factor-*α* (TNF-*α*), receptor activator of NF-*κ*B ligand (RANKL), and interleukin-1*β* (IL-1*β*). An in vivo ubiquitination assay was performed using endogenous immunoprecipitation of TRAF6 in bone marrow macrophages (BMMs) and osteoclasts.

**Results:**

Here, we report on a form of TRAF6 ubiquitination that is mediated by c-Cbl, leading to the formation of lysine 48-linked polyubiquitin chains. The NF-*κ*B activity induced by RANKL and IL-1*β* treatment is inhibited when c-Cbl is overexpressed, while the NF-*κ*B activity induced by TNFα treatment is not. c-Cbl inhibits NF-*κ*B activity mediated by TRAF6, but not by TRAF2. These findings show that c-Cbl ubiquitin ligase activity is essential for TRAF6 ubiquitination and negative regulation of NF-*κ*B activity. Fine mapping revealed that the proline-rich domain of c-Cbl is critical for interaction with TRAF6. Stimulation with RANKL or interferon-*γ* (IFN-*γ*) caused c-Cbl to bind to polyubiquitinated TRAF6.

**Conclusions:**

These findings indicate that the interaction of TRAF6 with c-Cbl causes lysine 48-linked polyubiquitination for both negative feedback regulation and signaling cross-talk between RANKL and IFN-*γ*.

## Background

Multiple components of nuclear factor *κ*-light-chain-enhancer of activated B cells (NF-*κ*B) signaling have been revealed to be ubiquitin ligases or substrates that regulate NF-*κ*B activation [1]. Regulatory ubiquitination is mediated by Lys63-linked polyubiquitination [2]. Tumor necrosis factor receptor-associated factor 6 (TRAF6) mediates NF-*κ*B activation, which affects a wide variety of cellular mechanisms. This signal has been implicated in inflammation, immune regulation, bone homeostasis, and development.

TRAF6 is a member of the TNF receptor-associated factor (TRAF) family. These proteins mediate signaling of the TNF receptor (TNFR) family, IL-1 receptor (IL-1R), IL-18 receptor (IL-18R), and Toll-like receptors. There are six known mammalian TRAF proteins that show direct or indirect interaction with members of the TNFR superfamily [3]. They all share a highly conserved c-terminal domain that is responsible for the interactions between TRAFs or interactions with other proteins. The N-terminal domain of TRAFs, which consists of one or more zinc finger domains, enables the activation of signaling cascades.

The RING domain of TRAF6 facilitates the synthesis of lysine 63-linked polyubiquitin chains through the unique E2 complex ubc13/Uev1A. Lysine 63 polyubiquitination of TRAF6 mediates the activation of the transforming growth factor beta-activated kinase 1 (TAK1) kinase complex, which subsequently activates IκB kinase (IKK) through phosphorylation of key serine residues within the activation loop of IKK*β* [2]. In vitro biochemical studies have suggested that the RING finger of TRAF6 ubiquitinates itself leading the IKK complex as an E3 ubiquitin ligase. TRAF6 ubiqitination through lysine 63-linked ubiquitin conjugation activates NF-*κ*B signaling, and it is the proteasome-independent pathway [2, 4].

The Cbl family comprises four members: c-Cbl, Cbl-b, *Caenorhabditis elegans* cbl (sli-1) and *Drosophila* cbl (D-cbl) [5]. Of these, c-Cbl and Cbl-b play prominent roles in the negative regulation of signaling from receptor tyrosine kinases [6–9]. Specifically, they mediate the degradation of activated signaling molecules in a RING finger-dependent manner [10–12]. c-Cbl is a cytoplasmic 120-kDa protein that consists of an SH2 domain, RING finger domain, proline-rich domain, and a leucine zipper [13, 14]. One of the established functions of the RING finger domain is the ability to mediate ubiquitination of other proteins by acting as an E3 ubiquitin ligase.

Previous studies have shown that c-Cbl is associated with TRAF6, but their precise relationship remains unknown [15]. In this study, we examined the ability of c-Cbl to ubiquitinate TRAF6 via stimulation of RANKL and IFN-*γ*. Such molecular and biochemical experiments based on NF-*κ*B signaling will lead to better understanding of the physiological processes involved in inflammation and immunity.

## Methods

### Plasmids, antibodies and reagents

The NF-*κ*B reporter vector (*κ*B)_3_-interferon-luciferase and pCMV-*β*-gal plasmids were previously described [16]. Expression constructs encoding RANK, TRAF2 and TRAF6 have also been described [17–19]. c-Cbl, CblG306E, CblC3AHN, and Cbl-1-655, − 1-480, − 1-436, − 1-421 and − 1-357 expression vectors were provided by Dr. H. Band [20, 21]. Ub, UbK63R and UbK48R expression vectors were provided by Dr. J.H. Kehrl [18]. The antibody (Ab) specific for ubiquitin was obtained from Chemicon; anti-TRAF6 (H-274) and anti-cbl (C-15) Abs were from Santa Cruz Biotechnology; anti-FLAG epitope Ab (M2) was from Sigma; and anti-HA epitope Ab was from Roche. Recombinant CSF-1, TNF-*α,* and IL-1*β* were purchased from R&D Systems; MG132 was purchased from Calbiochem; and soluble hCD8-RANK was purified from insect cells as previously described [19].

### Cell culture, cell stimulation, transfection, and luciferase assay

Osteoclasts were generated from bone marrow precursors as previously described [17], in which > 95% of adherent cells were ostoclasts. In vitro osteoclasts were extensively washed to remove exogenous growth factors, cultured in OPTI-MEM (GIBCO BRL) for 6 h, and then stimulated by adding the indicated cytokines. After stimulation, cells were washed in ice-cold phosphate-buffered saline (PBS), lysed, and subjected to western blot analysis or immunoprecipitation as described below.

Human embryonic kidney 293 T cells were cultured in Dulbecco’s modified Eagle medium (DMEM; Invitrogen Life Technologies) supplemented with 10% (v/v) fetal bovine serum (FBS; Gibco) and antibiotics. For transfection, Lipofectamine 2000 (Thermo Fisher Scientific Inc.) was used according to the manufacturer’s instructions. At 36 h post-transfection, the cells were harvested and whole cell extracts were prepared for the luciferase assay. The luciferase activity was measured using the Luciferase Assay System (Promega) and normalized relative to *β*-galactosidase activity as previously described [17]. Data were obtained from three independent transfections and are presented as the fold increase in luciferase activity (means ± SD) relative to the control.

### Immunoprecipitations and western blot analysis

To examine protein–protein interactions in cultured 293 T cells, subconfluent plates were transfected with 2–7 μg of the indicated combinations of expression vectors. At 36 h post-transfection, cells were lysed in 0.5% Nonidet P-40 lysis buffer consisting of 50 mM Tris-HCl (pH 8.0), 150 mM NaCl, 1 mM EDTA, 0.5% Nonidet P-40 and protease inhibitors. Cell lysates were incubated with anti-FLAG Ab, anti-HA Ab or anti-CBL Ab for 2 h at 4 °C and immune complexes were collected by incubation (1 h at 4 °C) with protein G-agarose (Roche Applied Science). After extensive washing, immunoprecipitated proteins were resolved using 6–10% SDS-PAGE and analyzed using western blotting with anti-FLAG, anti-HA, anti-Ub, anti-TRAF6 or anti-CBL Abs. For endogenous immunoprecipitation, bone marrow macrophages (BMMs) and osteoclasts were lysed in 0.5% Nonidet P-40 lysis buffer and incubated with polyclonal anti-CBL Ab or anti-TRAF6. Western blotting to detect endogenous TRAF6 and CBL was performed using polyclonal anti-TRAF6 and anti-CBL Abs, respectively.

### In vitro ubiquitination assays

Ubiquitination assays were performed in 2 μl reaction volumes containing the following components: 2 μg ubiquitin, 0.2 μg E1, 1.0 μg UbcH7 (Boston Biochem), 1 μgHA-purified c-Cbl, 0.2 μl recombinant TRAF6 and 2.5 μl 10X reaction buffer consisting of 300 mM HEPES (pH 7.2), 20 mM ATP, 50 mM MgCl_2_ and 2 mM DTT. Reactions were incubated at 30 °C for 1 h, terminated by the addition of sample buffer, and immediately heated to 95 °C for 5 min. Where indicated, immunoprecipitates were incubated with 2 μg TRAF6 or HA antibody for 1 h at 4 °C. Proteins were separated on SDS-polyacrylamide gels and then immunoblotted with the indicated antibodies.

### Statistical analysis

The results are expressed as the means ± standard deviation (SD) from at least three independent experiments. Two-tailed Student’s *t*-tests were used to analyze differences between groups. A *p* value less than 0.05/*p* < 0.05 was considered statistically significant.

## Results

### C-Cbl promotes TRAF6 ubiquitination and inhibits TRAF6-mediated NF-κB activation

TRAF6 is degraded by RANKL stimuli, and degradation of TRAF6 is protected by the proteasome inhibitor MG132 [21–23]. c-Cbl is a known interacting partner of TRAF6 [5, 15, 19] and the famous RING-type E3 ligase in receptor tyrosine kinase signaling, and has recently been reported in other signaling systems [10, 12, 20, 23].

**Fig. 1:**
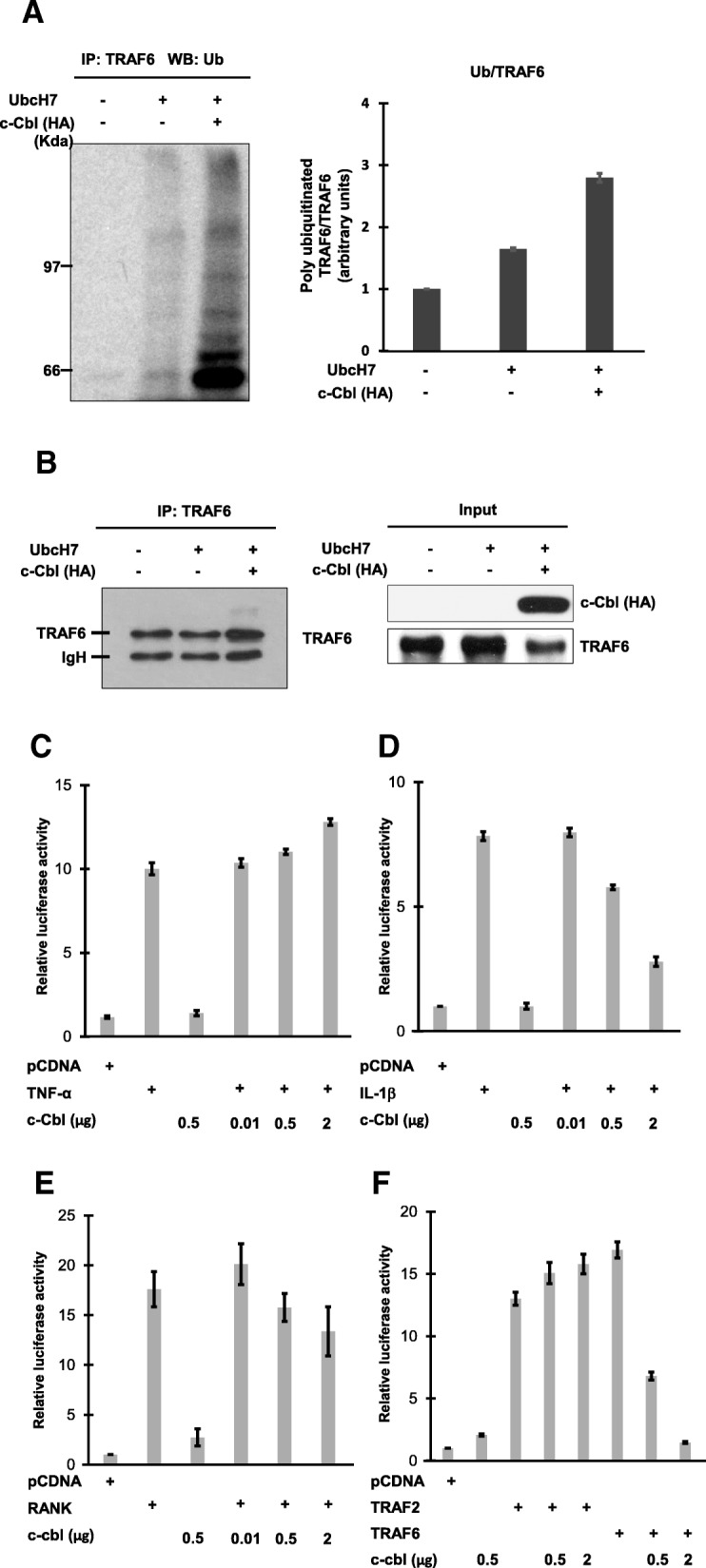
c-Cbl promotes TRAF6 ubiquitination and inhibits TRAF6 mediated NF-*κ*B activation. **a** – In vitro ubiquitination assays with immunoprecipitated TRAF6 from 293 T cells, transfected with either c-Cbl or an empty vector, and UbcH7 as ubiquitin-conjugating (E2) enzymes. These proteins were added to ubiquitination reactions consisting of E1, ATP and Ub, as described in the *Methods* section. Ubiquitinated proteins were detected by immunoblotting with anti-ubiquitin antibody. Quantification of the TRAF6 ubiquitination level of Ub/TRAF6. **b** – Immunoprecipitation of TRAF6 followed by immunoblot analysis with anti-TRAF6 (left panel) is shown. Controls for the expressions of HA-cbl and TRAF6 are shown (right panel). **c** and **d** – c-Cbl inhibited IL-1β-induced NF-*κ*B activation, but not TNF-*α* mediated NF-*κ*B activation. 293 T cells were co-transfected with 50 ng of (*κ*B)_3_-interferon–luciferase reporter plasmid, 25 ng of CMV-*β*-galactosidase plasmid, and 1 μg of pcDNA empty vector or HA-tagged pcDNA-c-Cbl, as indicated. 36 h after transfection, cells were treated with TNF-*α* (20 ng/ml) (**c**) or IL-1*β* (10 ng/ml) (**d**) for 6 h or left untreated. **e** – c-Cbl inhibited RANK mediated NF-*κ*B activation. 293 T cells were co-transfected with reporter plasmids and either a plasmid encoding RANK alone or c-cbl plasmid (1 μg), as indicated. **f** – c-Cbl inhibited TRAF6, but not TRAF2, and mediated NF-*k*B activation. Expression plasmids encoding TRAF2 and TRAF6 were tested for NF-*k*B activity toward c-Cbl. The results shown in (**c** through **f**) represent the means ± SD of triplicate experiments

To test whether c-Cbl could promote TRAF6 ubiquitination, TRAF6 was immunoprecipitated from 293 T cells transfected with c-Cbl or controls. IP beads were added to the in vitro ubiquitination assays. TRAF6 was ubiquitinated with UbcH7, which is a specific ubiquitin-conjugating (E2) enzyme for c-Cbl (Fig. 1a and b) [16]. Immunoprecipitated TRAF6 from the cells overexpressing c-Cbl showed strong ubiquitination (Fig. 1a and b).

TNF-*α*, RANKL, and IL-1*β* are typical stimuli of TRAF-mediated signaling pathways. Since TRAF RING-dependent ubiquitination is involved in NF-*κ*B activation, we examined whether c-Cbl-mediated ubiquitination affected NF-*κ*B activation following various stimuli of TRAF-mediated signals. 293 T cells were co-transfected with NF-*κ*B luciferase reporter plasmids either with empty vectors or c-Cbl expression plasmids. At 24 h post-transfection, cells were left untreated or treated with human TNF-*α* (20 ng/ml) or IL-1*β* (10 ng/ml) for 12 h. NF-*κ*B activity was then analyzed (Fig. 1c and d). The findings show that c-Cbl inhibited IL-1*β*-mediated NF-*κ*B activation in a dose-dependent manner (Fig. 1d) and inhibited RANK-mediated NF-*κ*B activation (Fig. 1e). Furthermore, expression of TRAF6 alone markedly increased NF-*κ*B activity in TRAF6-expressing cells, and co-expression of c-Cbl showed a dose-dependent decrease (Fig. 1f).

Next, we tested whether c-Cbl affected TRAF2-mediated NF-*κ*B activation, as TRAF2 shares a common motif with TRAF6 and TRAF2 ubiquitination is known to activate NF-*κ*B [24]. Overexpression of c-Cbl and TRAF2 did not repress NF-*κ*B activation, in contrast to the results for TRAF6 (Fig. 1f). Furthermore, TNF-*α*-treated cells showed NF-*κ*B activation regardless of c-Cbl overexpression (Fig. 1c). These data indicate that c-Cbl suppression of NF-*κ*B activity may be TRAF6 dependent.

### The Cbl RING finger domain is required for inhibition of TRAF6-mediated NF-κB activation

**Fig. 2:**
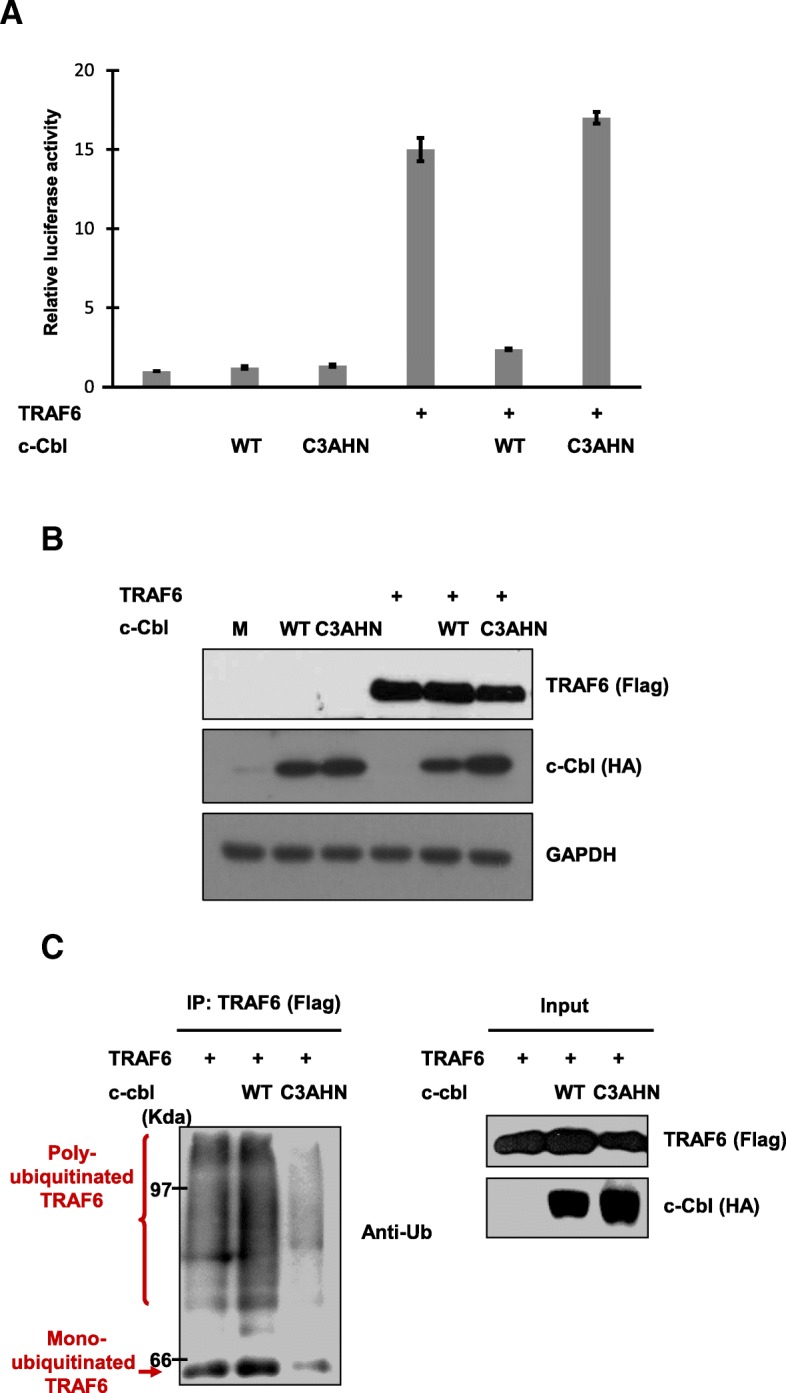
c-Cbl ubiquitinates TRAF6 via lysine 48-linked polyubiquitin conjugation dependent on the Cbl RING finger domain. **a** – c-Cbl C3AHN, the RING finger mutant, failed to repress TRAF6-mediated NF-*κ*B activation. 293 T cells were transfected with (*κ*B)_3_-interferon-luciferase reporter plasmid and the indicated combinations of Flag-TRAF6 (0.5 μg), HA-cbl (1 μg), and HA-cbl-C3AHN (1 μg) or pCDNA3.1 (+). The results are the means ± SD of triplicate experiments. **b** – Controls for ethe xpression of FLAG-TRAF6 and HA-c-Cbl are shown. GAPDH is used as a loading control. **c** – TRAF6 ubiquitination required the RING domain of c-Cbl. 293 T cells were transfected with the indicated combinations of expression plasmids. After 36 h of transfection, cells were treated with MG132 for 2 h and lysed. Cell lysates were immunoprecipitated with anti-FLAG Ab and then immunoblotted with anti-ubiquitin (ub). Controls for the expression of FLAG-TRAF6 and HA-c-Cbl are shown (right panels). **d** – 293 T cells were co-transfected with Flag-TRAF6 (1.0 μg), His-Ub (2 μg), and HA-cbl (1.0 μg) as indicated. After 36 h of transfection, cell lysates were immunoprecipitated with anti-FLAG Ab and immunoblottted with anti-ubiquitin. For expression controls, whole cell extracts were subjected to immunoblot analysis with anti-FLAG, anti-HA and anti-ubiquitin as indicated

To investigate the functional implications of Cbl-mediated TRAF6 ubiquitination, we compared the effects of c-Cbl and its RING finger mutant on TRAF6-mediated NF-*κ*B activation. TRAF6-induced NF-*κ*B activity was strongly suppressed with co-expression of c-Cbl (Fig. 2a). Interestingly, co-expression of the Cbl RING finger mutant failed to repress NF-*κ*B activity; rather, it enhanced activity to a certain extent (Fig. 2a and b). The loss of the ability to repress NF-*κ*B activity suggests that c-Cbl ubiquitinates TRAF6 and downregulates NF-*κ*B activation via RING finger domain-mediated Ub ligase activity.

Next, we directly tested the ability of c-Cbl and the RING finger mutant to target TRAF6 for ubiquitination in transfected 293 T cells (Fig. 2c). Until now, TRAF6 ubiquitination has been associated with lysine 63-linked polyubiquitination chains, which conjugate TRAF6 to elements upstream of NF-*κ*B [2, 21, 25]. Another form of lysine 48 polyubiquitination chains is the principal signal for proteolysis by proteasomes [26]. To clarify which polyubiquitination chain mediates this ubiquitination, 293 T cells were transfected with TRAF6, c-Cbl and/or ubiquitin including WT, K63R or K48R Ub, as indicated. In the case of co-transfection of c-Cbl, Cbl-mediated ubiquitination of TRAF6 was exclusively dependent on lysine 48-linked polyubiquitin chains (Fig. 2d).

### The RING finger and proline-rich domains of c-Cbl are required for interactions with TRAF6

**Fig. 3:**
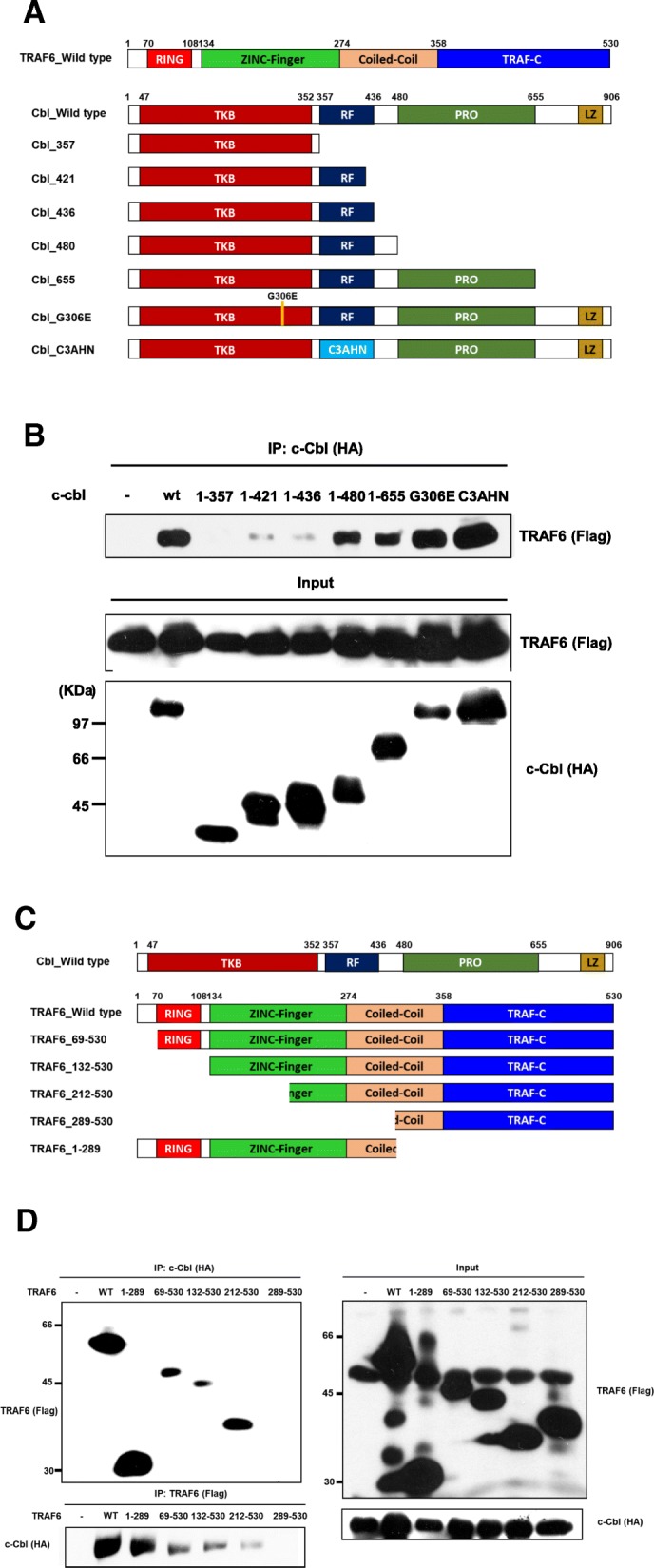
c-Cbl interacts with TRAF6 through the c-terminus of c-Cbl and N-terminus of TRAF6. **a** – Schematic illustration of wild-type TRAF6, wild-type c-Cbl and its mutants: *TKB*, tyrosine kinase-binding domain; *RF*, RING finger domain; *PRO*, proline-rich region; and *LZ*, leucine zipper. The number for each mutant indicates the Cbl residue that constitutes the c-terminus in the mutant protein. **b** – 293 T cells were transfected with FLAG-tagged TRAF6 (2 μg) together with expression plasmids encoding HA-tagged wild-type Cbl (*WT*), Cbl-G306E mutant (*G306E*), Cbl-C3AHN mutant, or various truncation mutants of Cbl, as indicated. After 36 h of transfection, cell lysates were immunoprecipitated with anti-HA Ab and then probed with anti-FLAG-TRAF6. Controls for the expression of FLAG-TRAF6 and HA-cbl are shown (middle and bottom panels). **c** – A schematic illustration of wild-type c-Cbl, wild-type TRAF6 and its mutants: RING, RING finger domain; and Zn FINGER, Zinc finger domain. **d** – 293 T cells were transfected with HA-tagged c-Cbl (2 μg) together with expression plasmids encoding FLAG-tagged wild-type TRAF6 (*WT*) or various truncation mutants of TRAF6, as indicated. After 36 h of transfection, cell lysates were immunoprecipitated with anti-HA or anti-FLAG Abs and then probed with anti-HA or anti-FLAG, respectively (left panels). Controls for the expression of FLAG-TRAF6 and HA-c-Cbl are shown (right panels)

The major pathway for receptor ubiquitination requires interaction between the SH2 domain of c-Cbl and tyrosine phosphorylation of the receptor [9, 11, 20, 23, 27]. Therefore, we examined c-Cbl to determine what domains are necessary for interaction with TRAF6. The following truncated and mutagenesis mutants were used: c-Cbl G306E, whose SH2 domain is defective; c-Cbl C3AHN, whose RING finger is defective; and truncated mutants lacking the proline-rich RING or SH2 domains (Fig. 3a). TRAF6 failed to interact with HA c-Cbl-1-357, which has an intact SH2 domain but lacks the RING finger (Fig. 3b). Thus, the presence of the SH2 domain is not critical for recruitment of TRAF6 to c-Cbl. By using the G306E mutant, we confirmed that the SH2 domain of c-Cbl is not involved in this interaction (Fig. 3b). TRAF6 associated strongly with HA-c-Cbl 1–655, which contains a proline-rich domain (Fig. 3b). HA c-Cbl 1–421, 1–436 and 1–480 have RING and SH2 domains. HA c-Cbl 1–421 and 1–436 showed weak interactions while HA c-Cbl 1–480 showed a relatively strong interaction, implying that an intact RING domain and additional 436–480 residues are crucial for their interactions (Fig. 3b). The RING finger was required for the interaction between TRAF6 and c-Cbl, but the C3AHN mutant still showed strong interaction with TRAF6 (Fig. 3b). We also examined the domains of TRAF6 necessary for interaction with c-Cbl (Fig. 3c). c-Cbl associated with Flag-TRAF6–1-289, − 69-530, − 132-530 and − 212-530, but not with − 289-530 (Fig. 3d). These results suggest that amino acid residues 212–289 contain the major interacting site (Fig. 3d).

### In vivo ubiquitination of endogenous TRAF6 in response to RANKL and IFN-γ stimuli

**Fig. 4:**
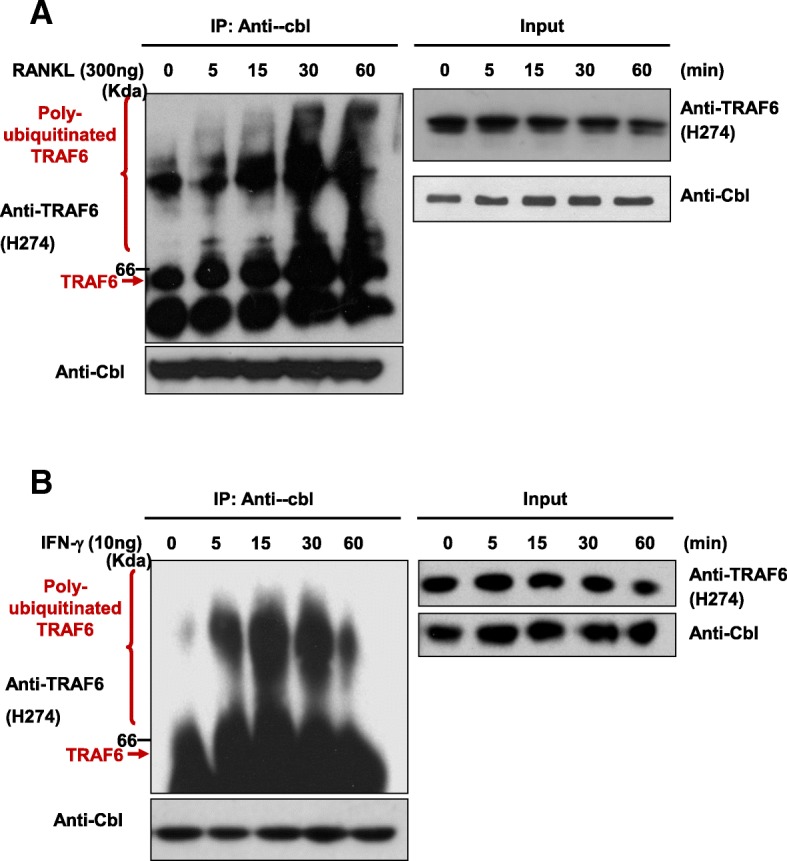
c-Cbl interacted with ubiquitinated TRAF6 after stimulation with RANKL and IFN-*γ*. **a** – Osteoclasts differentiated from bone marrow precursors were stimulated with RANKL for the indicated times and lysed. Cell extracts were immunoprecipitated with anti-cbl Ab and then probed with anti-TRAF6. The presence of endogenous TRAF6 or c-Cbl was analyzed via western blotting using anti-TRAF6 and anti-cbl. **b** – BMMs were extracted, as described in the *Methods* section, and treated with M-CSF (30 ng/ml). After three days, cells were stimulated with IFN-*γ* as indicated. Cell extracts were immunoprecipitated with anti-cbl Ab and then probed with anti-TRAF6. The presence of endogenous TRAF6 or c-Cbl was analyzed via western blotting using anti-TRAF6 and anti-cbl, as indicated

We tested whether endogenous Cbl interacted with polyubiquitinated TRAF6 in intact cells in a stimulation-dependent manner as a negative feedback regulation. Cell lysates were immunoprecipitated with anti-Cbl and then probed with anti-TRAF6. As shown in Fig. 4a, polyubiquitinated TRAF6 interacted with c-Cbl in a RANKL-stimulated manner, while endogenous TRAF6 was degraded by the stimuli. These findings are consistent with the idea that the interaction between c-Cbl and TRAF6 mediates downregulation of activated signals by TRAF6 ubiquitination.

Activated T cells are known to affect osteoclastogenesis [22], although the mechanism is unknown. Our results demonstrated that Cbl directly regulated TRAF6 ubiquitination via lysine 48 polyubiquitin chains. These findings could explain the signaling cross-talk between RANKL and IFN-*γ*: c-Cbl activated by IFN-*γ* could interact with TRAF6 and then induce ubiquitination.

To determine whether IFN-*γ* promoted TRAF6 degradation through c-Cbl-dependent ubiquitination, we administered IFN-*γ* treatment (20 ng/ml) at the indicated time points in BMMs. c-Cbl showed strong interaction with TRAF6 and the level of polyubiquitinated TRAF6 reached its maximum 15 min after stimulation with IFN-*γ* (Fig. 4b). Furthermore, TRAF6 western blot analysis showed larger smear bands after IFN-*γ* treatment, suggesting that c-Cbl recruits and ubiquitinates TRAF6 (Fig. 4b). From these results, we concluded that TRAF6 is degraded by c-Cbl-mediated ubiquitination and that this ubiquitination is necessary for regulating various stimuli.

## Discussion

TRAF6 is an essential protein involved in immune and inflammatory signaling pathways [28, 29]. Previous studies have shown that TRAF6 has ubiquitin E3 ligase activity in its RING domain. This mediates proteasome-independent ubiquitination via lysine 63 of ubiquitin [2, 21].

However, cells treated with MG132 showed enhanced TRAF6 ubiquitination, which implies that ubiquitinated TRAF6 is targeted for proteasomal degradation. Consistent with this finding, TRAF6 degradation is reportedly induced during RANKL and enhanced by ligation of the receptor for IFN-*γ* [22]. This suggests that the ubiquitin–proteasome system involving PA28 affects the degradation of TRAF6.

These findings led us to investigate whether TRAF6 could be targeted in the negative regulation of NF-*κ*B transcription through ubiquitination and proteasome-dependent degradation. Using both in vitro and in vivo ubiquitination assays, we showed that c-Cbl promoted TRAF6 ubiquitination by acting as an E3 ubiquitin ligase. When c-Cbl was overexpressed, the transcriptional activity of NF-*κ*B was significantly suppressed and this was followed by the stimulation of TRAF6-mediated signals/and TRAF6-mediated signals were stimulated. Despite the interaction with TRAF6, the RING finger mutant of c-Cbl failed to suppress both NF-*κ*B activation and ubiquitination promotion.

These results suggest that c-Cbl downregulates NF-*κ*B signaling by catalyzing TRAF6 ubiquitination. The RING domain of c-Cbl is required for the E3 ligase activity involved in this process. Consistent with this hypothesis, the interaction of polyubiquitinated TRAF6 with c-Cbl gradually increases after RANKL treatment, while the total amounts of TRAF6 protein diminish over time.

To form polyubiquitin chains, isopeptide bonds between the lysine residue of one ubiquitin and the c-terminal G76 of another ubiquitin are needed [30]. In general, lysine 48-linked polyubiquitin chains are recognized by proteasomes, while lysine 63-linked chains are not likely to involve proteasomes. TRAF6 contains a RING domain that facilitates the synthesis of lysine 63-linked polyubiquitin chains by Ubc13/Uev1A. Co-expression of ubiquitin mutants with lysine 48R and lysine 63R demonstrated that TRAF6 was exclusively ubiquitinated with lysine 48-linked polyubiquitin chains when co-expressed with c-Cbl, suggesting that c-Cbl promotes TRAF6 degradation.

Therefore, we propose that TRAF6 ubiquitination is involved in NF-*κ*B activation and its negative regulation. TRAF6 initially activates the TAK1 kinase complex through the lysine 63 polyubiquitin chain, and this step requires TRAF6 as an E3 ligase. As a result, TRAF6 is degraded by direct c-Cbl ubiquitination via the lysine 48 polyubiquitin chain.

c-Cbl comprises a conserved phosphotyrosine-binding domain (PTB), a small zinc-binding domain known as the RING finger domain, a proline-rich region, and multiple tyrosine phosphorylation sites. In ubiquitination, the PTB domain binds to specific phosphotyrosine sites and the RING finger domain recruits the ubiquitin conjugating enzyme UbcH7. In our experiments, c-Cbl G306E mutants were still able to interact with TRAF6, suggesting that tyrosine phosphorylation is not required for this interaction. Since the TRAF6-c-Cbl association was induced by RANKL, this ubiquitination may constitute negative feedback regulation of RANKL signaling.

It has been reported that activated T cells produce IFN-*γ* and negatively regulate osteoclastogenesis [22]. Interestingly, c-Cbl interacted with ubiquitinated TRAF6 following engagement of IFN-*γ* in BMMs. Although TRAF6 ubiquitination is reportedly mediated by the lysine 63-linked polyubiquitin chain, our results suggest that TRAF6 ubiquitination requires both the lysine 48 and lysine 63 residues of ubiquitin [31]. These findings suggest that the combined ubiquitin system is necessary for controlling various stimuli related to negative feedback regulation or cross-talk.

## Abbreviations

BMM: Bone marrow macrophages
IFN-*γ*: Interferon-*γ*
IKK: IκB kinase
IL-18R: IL-18 receptor
IL-1R: IL-1 receptor
IL-1*β*: Interleukin-1*β*
NF-*κ*B: Nuclear factor *κ*-light-chain-enhancer of activated B cells
RANKL: Receptor activator of NF-κB ligand
TAK1: Transforming growth factor beta-activated kinase 1
TNFR: TNF receptor
TNF-*α*: Tumor necrosis factor-*α*
TRAF6: Tumor necrosis factor receptor-associated factor 6
